# Chicago Medical Society

**Published:** 1885-01

**Authors:** 


CHICAGO MEDICAL SOCIETY.

*Meeting of December 1, 1884.*—The President, Dr. D. A. K. Steele, in the chair; Dr. Liston H. Montgomery, Secretary.

At the semi-monthly meeting, held on the evening of December 1, 1884, the regular order of scientific and other important business was, upon motion, temporarily suspended, and the following appropriate remarks and resolutions of respect relative to the death of Dr. Abram Groesbeck, an old and well-known physician and fellow of the Society, whose demise occurred November 25, 1884, at the age of 74 years, 7 months and 15 days, were introduced by Dr. R. C. Hamill and unanimously adopted.

*Mr. President and Fellow Members of the Chicago Medical Society:*—I arise to offer a tribute of respect to the memory of a beloved member of this Society, who, in the Providence of God, has been suddenly summoned from our midst to appear at the council chamber of the Great Physician.

Death has made many reprisals from the ranks of our profession in these last years. Some who had just entered the portals of professional life, with bright prospects of eminent success in full view, have been cut off in the very flower of their days—others in the vigor and maturity of their years, manfully bearing their part in the battle of life, have fallen in full clad armor in the front ranks—but he to whom I call your attention to-night had more than filled his cycle of three score years and ten, and a half century of that time had been devoted to professional services at the bedside of the sick and suffering.

> "The leaves of the oak and willow shall fade,
> Be scattered around and together be laid;
> And the young and the old, and the low and the high,
> Shall moulder to dust and together shall lie.
>
> * * * *
>
> "The hand of the King that the sceptre hath borne,
> The brow of the Priest that the mitre had worn:
> The eyes of the Sage and the heart of the brave,
> Are hidden and lost in the depth of the grave."

Dr. Abram Groesbeck was no common man, but a learned and scientific Doctor of Medicine, and a man of decided character, as fellows present, who have had a more intimate association with him than has been my fortune, in the remarks that are to follow, I am sure will take great pleasure in declaring.

He lived a life of usefulness, and the world is better for such living—he died in the fullness of his years, his intellect unclouded and his love for the literature of the profession as ardent as in the vigor of his days, an honor to the cause to which the energies of a well-spent life had been devoted, respected by his fellow practitioners, revered and affectionately beloved by the families who for so many years have enjoyed the benefits of his professional skill.

WHEREAS, This Association is called to mourn the loss of one of its beloved members, who, for more than a quarter of a century has lived the life of an honorable and successful physician in our city;

*Resolved*, That we contemplate with great pleasure the well-rounded character of Dr. Abram Groesbeck, who has so faithfully filled the measure of a true American physician, and commend his example as a Christian and a physician to those of our number who are entering upon the arduous and responsible duties of our profession. We regret that we are called upon to part with him forever—that we shall meet him no more at the bedside of suffering—in the social circle or in the busy walks of daily life.

*Resolved,* That while we regret to note upon the records of our Society the demise of our greatly respected fellow, yet it is with pleasure we contemplate the fact that up to the final summons his mental faculties remained in full strength, and that he was able to contemplate with perfect calmness the approaching catastrophe, saying in that prompt and decisive manner so characteristic of him: "I have accomplished my mission here, and it is better to go now than suffer the pains and aches incident to extreme age."

*Resolved,* That we admire the learned and gentlemanly physician, and cherish the example he left us as a rich inheritance, and claim the privilege of dropping a tear of sincere sympathy with the bereaved family in this the day of their sorrow.

*Resolved,* That a copy of these resolutions be sent to the family of the deceased by the Secretary of this Society.

Dr. John Bartlett spoke as follows:

*Mr. President:*—I desire to say a few words in memoriam of the friend and fellow from whom death has just separated us.

Dr. Abram Groesbeck was born at Albany, N.Y., May 24, 1810. He began his medical studies in his native city, becoming a licentiate of the New York State Medical Society in 1831, and graduating at Albany in 1849. He settled in Chicago in 1856, and continued to practice medicine actively till a few years since, retiring only when he was no longer able to see to write a prescription. It was a circumstance which rendered the very last days of his life happier that "loosed from his environment" in part, as he was by impairment of sight and hearing, and other infirmities, he yet had a few patients to visit. For some years past symptoms of angina pectoris had developed, and two months since he had a violent attack, from which, however, he recovered, and he was in his usual health till the 24th ult., when he was taken without pain with Cheyne-Stoke's respiration, the period of apnœa alternating with rapid breathing each minute. Meanwhile the action of the heart grew feebler for eighteen hours, when unconsciousness supervened, soon followed by death. Dr. Groesbeck was fully aware of his condition, and yet he spoke cheerily to all, not omitting jocosely to rally the profession upon its want of understanding of, and lack of cure for, his ailment. The doctor expressed himself as prepared and willing to depart this life, declaring that his work was done.

He was a member of the Episcopal church.

Dr. Groesbeck had a tall and graceful figure and a fine face. His features strikingly resembled those of the late Dr. Gross. In manner he was a gentleman of the old school—genial, courteous, amiable. He was of a kindly spirit and benevolent disposition, his most prominent characteristic being sacrifice of self for the advantage of others.

Dr. Groesbeck was endowed with a superior intellect. He received a classical education which led to an appreciation of good literature and the cultivation of excellent taste for the best writers, ancient and modern. His familiarity with the classics he retained in a remarkable degree. It was one of his amusements to confound a friend by leaving his compliments and message on his slate in the language of Celsus. He retained also his knowledge of the French. Even in the last months of his life he selected for reading such writings as those of Tacitus; and only recently he planned to have read to him again the Æneid of Virgil. It gave him great pleasure to exalt the matter and style of the ancients, and to credit them with knowledge, philosophy and rhetorical skill. He liked to dwell among the old moderns, and read with deep interest such works as Pepy's Diary. On one occasion he insisted on leading a medical friend into the Public Library, that he might there read how the physician of one of his favorite old diarists successfully prescribed, hundreds of years ago, milk diet as a cure for diseases of the kidneys. Of late years the doctor had been more interested in religious works, as in "Sceptical Fallacies," by W. J. Hall; and in the volume of Henry Drummond, "Natural Law in the Spiritual World." The latter production he pronounced remarkable.

"One of the saddest reflections of my later years," said recently one of the ablest of Chicago's medical men, "is that the many minor means and arts which experience has given me for the cure of disease and the relief of suffering will perish with me." Dr. Groesbeck was an old and skillful practitioner, and yet, so far as I am aware, he has left no record of his experience. It occurs to me as proper here to note a few of the views, which memory now recalls, held by him as the sum of his experience in certain departments of practice. However opposed the views of the doctor may prove to those commonly promulgated by the professional teacher, it is well to grant due weight to the opinions of so experienced and sagacious a practitioner. The doctor was by nature sceptical, and he was ever on his guard lest he might lend credence to some view not thoroughly established. While interested in theory, he was, preferring methods of true empiricism, loth to acknowledge its value. To the enthusiastic germ theorist who might occupy his attention with ingenious possibilities as to the cause of disease, he was ever ready with his chilling cry, "*cui bono!*" Long before the German cry, "*Fort mit dem spray*,"

was heard over the land, Dr. Groesbeck, when invited to witness an ovariotomy, satirically inquired, "Are they to *play* with the spray?" Dr. Groesbeck often declared himself a disciple of Dr. Todd of London, and for years past had treated pneumonia not as a phlegmasia, but as a form of fever; in some cases giving a bottle of wine in twenty-four hours with apparent advantage. Even the statistical information, furnished since the death of Dr. Todd, adverse to his ideas as to the value of the stimulant treatment, did not change the opinion or practice of Dr. Groesbeck.

Dr. Groesbeck had ever before him in the practice of his profession the strong influence of the mind over the body of the sick. He deemed it a part of the skill of the physician to protect his patient from anxieties and fear. His manner and converse in the sick-room, therefore, were such as to inspire hope and courage in those under his care. It may be stated that Dr. Groesbeck deemed it in practice the part of wisdom to avoid suggesting a consultation; and this, on the ground that it diminished the confidence of the patient in his attendant, and with confidence shaken, no small part of the power of treatment for good he believed to be lost.

For a number of years past the doctor was afflicted with a degree of deafness precluding anything like satisfactory auscultation. It was remarkable how little this circumstance lessened his confidence in diagnosing diseases of the chest. He trusted entirely to acute observation, and shrewd interpretation of the ordinary symptoms.

The doctor looked upon the placenta blocking the os uteri or vagina after labor as a natural and most useful tampon, and he regarded too early an interference with it as a mischievous interruption of nature in her efforts to close and occlude the uterine vessels. Naturally he agreed with Braithwaite, that in certain conditions of hemorrhage after labor, the uterus being contracted, to tampon was good practice; and he went so far as to suggest that probably in such cases the re-introduced placenta might be the best plug that the accoucheur could employ. The doctor had for nearly forty years of his practice an unusual number of cases of post-partum hemorrhage. He had meantime conducted the third stage of labor according to the standard precepts of the day. Of late years he had fewer cases of hemorrhage, and he referred the change in his experience to a change in his practice. As an almost invariable rule he had during the first decades of his work given ergot immediately after the birth of the child. It was following a discontinuance of this practice that the comparative freedom from post-partum bleeding occurred.

In placenta prævia centralis Dr. Groesbeck believed that perforation of placenta gave as good results as the passing of the hand between it and the uterus, and he regarded the former method as easier, and more speedy than the latter. When under any circumstances turning had to be resorted to, Dr. Groesbeck delivered promptly, impressing on the child the necessary turns, and never waiting for nature to do so. In breech presentations the doctor thought it better practice to break the wedge as soon as the os was sufficietly dilated, bringing down both feet and delivering at once.

The Doctor was an advocate of the rather frequent use of the forceps, using the old model Bedford for higher operations. When the head was lower in the pelvis he used the short forceps, always speaking highly of their convenience and efficiency. It should be remarked, however, that in the selection of the short instrument, Dr. Groesbeck rejected those in the construction of which the maker, in shortening the handles, shortened also the cephalic curve.

Dr. Groesbeck fully endorsed the views of the anatomy of the cervix uteri gravidi held by Braune, Bandl, Barnes and others. He believed that the placenta prævia was located below the os internum.

The doctor was possessed of an unusual degree of candor and was entirely void of professional pretense. He often admitted his shortcomings ; thus, he did not hesitate to state that the mechanism of labor as regards the mutations of the fœtal head in passing the paturient canal were to him inexplicable. He declared that he was generally unable to determine the position of the head, and that the reputed "planes and spines" effecting changes in it were to him a "terra incognita."

His favorite obstetrical work was "The Principles of Midwifery, by John Burns." In the preference here indicated our colleague complimented his own judgment as well as the classical production of the Glasgow professor.

The Doctor had a keen appreciation of the ways and means which tact and experience teach as aids to success in practice. In a knowledge of the subtle methods by which a physician may ingratiate himself with the people, magnify his importance, and obtain and retain practice, Dr. Groesbeck was not second to the author of "The Physician Himself." But while he was prodigal of advice in such matters to others, he took, in his own conduct, little heed of his own wisdom.

After he had been in the profession half a century he felt less confident that he would in any case do all that was possible for a patient than he had felt fifty years before. He on one occasion expressed surprise at the recollection of the nonchalant unconcern with which as a novice he had undertaken the management of an adherent placenta or placenta prævia.

As to the profession as a business, Dr. Groesbeck held an opinion contrary to that of the great mass of practitioners. He always insisted that the practice of medicine was upon the whole as easy and as pleasant a mode of earning a livelihood as any of which he had knowledge.

Dr. Groesbeck felt a fatherly interest in the younger members of the profession, and never tired of stimulating their thought and enlarging their knowledge by the narration of instructive cases and the enunciation of cardinal precepts.

Mr. President: From the shores of the mystical river we call to our vanishing friend the final word of mortals: Vale! Vale! An adieu laden with uncertainty, yet buoyant with hope. As we turn from the dark waters to the duties yet before us, let us pause to inscribe our index memorabilium with the honorable name of Abram Groesbeck.

*An Extensive Injury of the Face.*—Dr. Robert Tilley related the history of the case, and showed the patient, a boy about five years old, who had been injured by an elevator on the 23d of August. He was lying down on a floor, looking down an elevator shaft, when the platform descended and struck him on the back of the head, just beneath the occipital protuberance, forcing the bridge of the nose against the edge of the opening in the floor. The resistance of the parts at the back of the neck and head being relatively great, very little injury followed in that situation, but the greater part of the force of the blow was sustained by the face, upon which the edge of the floor had evidently impinged, just at the junction of the frontal, nasal, and superior maxillary bones, producing a star-shaped laceration of the soft parts running up the forehead about an inch from the root of the nose, downward along the bridge of the nose for about the same distance, then upward along the right side of the frontal bone to a point corresponding to the outer end of the eyebrow. These wounds extended through the soft parts to the bone, and two others radiated downward and outward from the root of the nose. The nasal bones were crushed and in part destroyed, the soft parts were torn from the bones, and the cartilaginous septum of the nose was wrenched from the bony septum. The nose and adjacent parts hung down over the mouth and the lower lip, and it appeared as if the right eye was destroyed.

Absorbent cotton soaked in dilute acetic acid was packed in between the soft parts and the superior maxillia, on each side, to stop the bleeding, and the assistance of Dr. Charles Adams, Dr. A. B. Hosmer, and subsequently Dr. C. S. Parkes, was procured. It was decided not to try to force the two maxillary bones closer together, but to leave the parts to heal by granulation, especially as it was thought that it would be difficult to anæsthetize the patient. It was questioned, too, whether the advantage gained would equal the additional discomfort from any device that could be adopted. The boy was in a semi-unconscious state, but there did not seem to be any special indication of brain lesion. The soft parts were brought into good apposition with stitches, and both eyes were occluded with a pressure bandage. There was a good deal of œdema about the right eye. Slight fever followed at once and increased considerably on the fourth day, but yielded readily to the use of warm baths and a little calomel. Up to the time of the removal of the pressure due to the bandage there was persistent somnolence, but, when the bandage was taken off, the boy said, as if in answer to a question, "The elevator struck me." At intervals after this he told the circumstances of the accident, and called to mind various objects he had seen in Dr. Tilley's office at the time the wounds were dressed. It was thought best not to reapply the pressure, and consequently the œdema increased, but union had taken place, except at the root of the nose, and all but two or three of the stitches were removed. Great swelling persisted, however, and subsequent interstitial suppuration made it necessary to reopen the superficial wound on the right side of the face and the superficial part of the deep wound on the left side. These two wounds were very obstinate in healing, and interstitial suppuration in the contused parts about the right eye had to be relieved by incision, which resulted in a scar at the malar prominence and the subsequent production of ectropion. The tendo oculi seemed to have been torn away from its attachment, causing the orbicularis palpebrarum to act at a corresponding disadvantage.

For nearly three months there was almost absolute anæsthesia of the parts of the face implicated. There was marked diplopia, but it was evidently due to paresis of the muscles of the right eye, and the speaker thought there was no disturbance of the relative position of the two eyeballs. The paresis was now much relieved, and the anæsthesia had almost disappeared, but the canaliculi were quite distorted, and it would probably be necessary to lay them open. The gap between the superior maxillary bones was completely closed, and the nasal passages had been almost occluded by adhesion of the inferior turbinated bones to the floor of the inferior meatus; but a passage had been forced through on each side with the galvanic cautery, and the boy's breathing and enunciation were now practically normal. It was proposed to remedy the ectropion and the general displacement of the right eyelids by loosening the cicatrix over the malar prominence as much as possible, and perhaps by making a counter-cicatrix on the inner side of the lid with the galvanic cautery; also try to make a substitute for the disorganized tendo oculi by causing the integuments to adhere to the bony structures opposite the inner canthus.

Dr. C. T. Parkes, who had seen the boy soon after the accident, thought that the result had been much better than could have been expected, in view of the terrible nature of the injury. Doubtless the measures which Dr. Tilley proposed would overcome the ectropion, which was the most prominent deformity that now remained.

Dr. W. W. Jaggard read a paper entitled "*Palliative Measures in Ruptured Extra-Uterine Pregnancy.*" An editorial bearing this title and appearing in the *New York Medical Record,* of Oct. 25, 1884, contains the following statement:

"There is no palliative measure for a ruptured extra-uterine cyst; there is no expectant treatment; and there is no other way known to medicine by which a woman in this condition can be reasonably expected to survive, save by the prompt use of the knife, and there is no reason for thinking that she would die if this be resorted to in time."

The object of this paper is to offer a protest against this *ex cathedrâ* mode of settling a question, in regard to which there is room for considerable latitude of opinion. Dogmatism, offensive and unphilosophical under all circumstances, attains its acme of arrogance when applied to rules of practice in medicine and surgery. Certainly, the use of the universal proposition, in the assertion just quoted, is both in bad taste and positively erroneous. In the ensuing discussion, attention is limited to the consideration of tubal pregnancy, because of its most frequent occurrence, and usually early termination. Furthermore, criticism of the treatment of the case, detailed in the *Record's* editorial, is incidental.

Tubal pregnancies may terminate: (1) in the death of the embryo and gradual absorption of the ovum, before rupture of the sac; (2) the sac may rupture, but the egg may remain within, and act as a tampon; (3) the cyst may rupture into the

*ligamentum latum*, with the formation of a hæmatoma ; (4) the sac may rupture into the peritoneal cavity, with the formation of a recto-uterine hæmatocele ; (5) the sac may rupture into the peritoneal cavity, and the life of the woman may be threatened by free hemorrhage, or the resulting peritonitis ; (6) the ectopic pregnancy may persist until the expiration of the full period of utero-gestation. It is impossible, in the present state of medical knowledge, to make any positive statement as to the relative frequency of these terminations. Until within a comparatively recent period of time, only those cases were designated tubal pregnancy in which the diagnosis was made by an autopsy. It is highly probable that ectopic gestation is of more frequent occurrence than the older systematic writers would lead one to believe. It is also probable that termination by recovery is not an uncommon event. These assertions receive some support from the experience of Professor Karl Schroeder :

* "I myself see so frequently cases of tubal pregnancy, in which the diagnosis is positive, pursuing a favorable course, that I consider recovery as the regular termination."

The second, third, fourth and fifth modes of termination are alone pertinent to the present discussion.

(2) *The sac may rupture, but the egg may remain within, and act as a tampon.* Such cases, with favorable results to the mothers, have been observed by *Wiedersperg* † and *Virchow.*

Operative interference in such cases is so obviously contraindicated that it is unnecessary to enter upon any comment upon that subject.

(3) *That cyst may rupture into the ligamentum latum, with the formation of a hæmatoma.*

---

* Karl Schroeder's Lahrbuch d. Geburtshülfe, Bonn, 1884, p. 422.

† Otto Spiegelberg's Lehrbuch d. Geburtshülfe, Lahr, 1882, p. 290.

This termination of tubal pregnancy has been observed, up to the present time, with comparative infrequency.* There can be no doubt, however, as to its actual occurrence. The sac ruptures into the broad ligament, the embryo escapes, and an extraperitoneal hæmatoma arises. The embryo may go on to full development in this region; usually it dies and undergoes reductive metamorphosis, while hemorrhage is checked by the pressure of the folds of the broad ligament.

Schuchardt † has demonstrated the possibility of this mode of termination by his celebrated case, published in Virchow's Archives. J. Veit ‡ has observed the same occurrence.

Primary laparotomy is not indicated by this termination, for the reason that the natural history of the condition shows that hemorrhage is usually controlled by the relations of the parts, and ultimately complete resorption of coagula and embryo occurs. Exceptional cases, however, may indicate operative procedure for the arrest of hemorrhage or removal of the embryo.

(4) *The sac may rupture and the fœtus escape into the peritoneal cavity, with the formation of a retro-uterine hæmatocele.*

Ollivier, Leclerc, Schroeder, Vignés, Gallard, Karl Braun, Veit, Chiari, and others, have observed this mode of termination. There can be no doubt as to its occurrence,

As to the frequency of its occurrence, opinions and statistics widely differ. Gallard || makes the statement that hæmatoceles, arising independently of trauma, are almost always due to the rupture of the cyst of extra-uterine pregnancy. Shroeder § says: "This etiology of the hemorrhage (retro-

* Karl Braun's Lehrbuch der Gesammten Gynækologie, Wien, 1883, p. 634.

† Virchow's Arch. Bd. 89, p. 133.

‡ Die Eileiterschwangerschaft—von Dr. J. Veit, Stuttgart, 1884.

|| Lecons cliniques des maladies des femmes. Paris, 1875, p. 635 ff.

§ Handbuch der Krankheiten der Weiblichen Geschlechts organe, von Dr. Karl Schroeder, Leipzig, 1881, p. 452.

uterine hæmatocele) is decidedly of very frequent occurrence, even if the tubal pregnancy is seldom diagnosticated."

Veit * has very recently collected 146 cases of hæmatocele, of which 40, or 28 per cent., are referred to this mode of origin. He is convinced that 28 per cent. is a low estimate of the frequency of the occurrence of this etiological factor. It is highly probable, if not positively determined, that the rupture of the cyst of ectopic pregnancy is a more frequent cause of retro-uterine hæmatocele than the standard American and English text-books are disposed to admit.

The course and results of retro-uterine hæmatocele, caused by the rupture of the cyst of extra-uterine pregnancy, have been the subjects of recent study. The extravasated blood is collected in the recto-uterine peritoneal pouch, encysted, and, together with the product of conception, may be resorbed after a very variable interval. Hemorrhage in these cases is arrested by the diminution in the force and frequency of the heart's action, the process of thrombosis, and the equalization of blood pressure within the arteries and the extravasation. (Schroeder.) Death † from anæmia, in the 40 cases collected by Veit, occurred but three times. No one who is at all familiar with the literature of the subject; more particularly with Leopold's experiments upon the lower animals, can entertain any doubt as to the complete resorption of the embryo. Schroeder has seen, in the autopsy of a woman, dying from a ruptured tubal pregnancy, all the peritoneal lymphatic vessels filled with red blood. Fatal peritonitis, acute or chronic, like fatal hemorrhage, is a relatively rare termination. Schroeder, Veit and Voisin have expressed themselves very positively in favor of the view that, after rupture of the cyst of tubal preg-

* Die Eileiterschwangerschaft von Dr. J. Veit. Stuttgart, 1884, p. 14.

† Die Eileitershwangerschaft, von Dr. J. Veit, Stuttgart, 1884, p. 15.

nancy, with the escape of product of conception and the formation of a retro-uterine hæmatocele, recovery is the rule, and death the exception.

The prognosis is the same as in cases of hæmatocele from other causes. Hemorrhage is seldom the cause of death. Peritonitis is a prognostic element of more serious import. Of the 40 cases collected by Veit, 11 died of peritonitis, at intervals of from a few days to several months.

Remarkable unanimity of opinion, as regards the treatment of retro-uterine hæmatocele, from whatever cause, exists at the present time. Schroeder, Veit, Karl Braun, Spiegelberg, Barnes, Emmet and Thomas advise the expectant plan ot treatment. Absolute rest in bed in the horizontal position, or with the hips elevated and the thighs slightly flexed upon the abdomen, the local application of cold,—ice-bags to the abdomen, bits of ice in the vagina, cold water rectal irrigation,—catheterization of the bladder, in case of retention of urine, the exhibition of opium and chloral hydrate, are points of treatment to which it is scarcely necessary to call attention in this connection.

Surgical interference, at an early stage, is seldom, if ever, indicated, for reasons detailed in the foregoing brief sketch of the natural course and results of the condition.

Surgical interference, at a later stage, is considered justifiable under two conditions: (1) persistence of the tumor in its original volume through weeks, without any diminution or alteration, with the occurrence of pains, which confine the patient to her bed for a considerable period of time; (2) the occurrence of suppuration or ichorous ulceration within the tumor. (Bandl, Billroth's Handbook.)

Even under these indications, laparotomy is not regarded as the operation of election. Dr. A. Martin astonished the members of the *Naturforscherversammlung*, in Salzburg, by the communication that he had performed laparotomy three times, on account of abdominal blood tumors. The three patients died. Baumgärtner, since the reading of Dr. Martin's paper, has performed laparotomy, on account of a peri-uterine blood tumor, once. The patient made an excellent recovery. With these statistics, laparotomy has roused no degree of enthusiism. Operators of the present day limit their interference to the various modifications of the methods originally suggested and practiced by Nélaton, that is, vaginal puncture, or incision.

(5) *The sac may rupture into the peritoneal cavity, and the life of the woman may be threatened by free hemorrhage, or the resulting peritonitis.*

Free hemorrhage into the peritoneal cavity and consecutive peritonitis have been regarded, for so long a period of time, as the exclusive mode of termination of tubal pregnancy that no evidence is required to establish the fact of occurrence. Again, opinions and statistics differ widely as to the relative frequency of this mode of termination. The tendency of modern investigation is in favor of the view that free hemorrhage into the peritoneal cavity and resulting peritonitis are unusual occurrences. * Fatal hemorrhage is very seldom observed, according to the observation of J. Veit, when rational expectant treatment has been practiced. Cases terminating by recovery either do not come under observation, or doubt as to the accuracy of the diagnosis is entertained. On the other hand, cases terminating by death from hemorrhage or peritonitis are almost always subjected to post-mortem examination. Very naturally, in the course of time, the fallacious induction—that death from hemorrhage or peritonitis is the rule—has been made.

---

* Die Eileitschwangerschaft, von Dr. J. Veit, Stuttgart, 1884, p. 65.

It is highly probable that many of the cases, recently reported in American and foreign journals, of favorable termination of tubal pregnancy before rupture of the cyst, as the result of the passage of the electric current, have resulted favorably, not from the death and resorption of the embryo within the sac, but from rupture of the cyst, death and escape of the embryo in the modes just indicated. The effect of an electric current upon smooth muscular fiber is to produce a contraction. When the number of séances, the currents employed, and the symptoms following, are taken into consideration, it is difficult to escape the conviction that in some cases, at least, the favorable result ought to be ascribed to rupture of the cyst. The question of treatment, when the patient's life is threatened by free hemorrhage into the peritoneal cavity, or by peritonitis, is diffiult and important.

Wiltshire, Lawson Tait, Knowlsley Thornton, and *our editorial friend* of the *New York Medical Record*, see in this condition an absolute indication for *immediate laparotomy*. There are others—and they may be justly designated "surgical leaders of the day"—who do not recognize this absolute indication for immediate operative procedure.

The prognosis of laparotomy, after rupture of the cyst, is by no means as favorable as in tubal pregnancy before rupture.

The reasons for gloomy prognosis are evident.

The operation must be performed upon a woman in a condition of more or less profound shock. The state of acute anæmia exercises an unfavorable influence. Ether is dangerous from the possibility of dislodgement of a thrombus and renewal of hemorrhage. Chloroform must be used with extreme caution, on account of the enfeebled heart. The blood poured out into the peritoneal cavity is mechanically removed, instead of undergoing absorption. The *technical* difficulties of the operation are great. The anatomical relations of the parts are very different from those in cases of consecutive or secondary hemorrhage, after extirpation of tumors by abdominal section. It is difficult, even on post-mortem examination, to differentiate between tissues and organs. *In viva*, the complications are still more intricate, as all who have had experience will testify. The choice of time, of place, and of operator, is out of the question. Abdominal section must be made without qualified assistants, and without attention to the principles of antiseptic surgery. If the patient is removed to a hospital, she may die on the way. If she survives transport, the assumption of spontaneous recovery is justifiable.

"Is, therefore," says Veit, "the prognosis of the operative procedure gloomy *per se*, the prognosis of the tubal pregnancy remains the same; indeed, it grows better with every hour the patient lives after rupture, so that of laparotomy and arrest of hemorrhage there really can be no serious talk." Finally, Veit, after a careful study of the literature of the subject, has been unable to find *one recorded case*, in which the patient's * life was saved by the operation.

Under the expectant plan of treatment is included a variety of expedients. Absolute rest in bed, in the horizontal position, the free exhibition of opium, compression of the abdominal aorta by sand-bags, shot-bags, ice-bags, tourniquet, or the hand, rectal irrigation with ice-water, are the more important methods by which hemorrhage may be controlled and the process of thrombosis favored. It is not necessary to add that all cardiac stimulants and counter-irritants must be proscribed.

If, notwithstanding this therapy, symptoms of internal

* Veit. Die Eileiterschangerschaft, p. 64.

hemorrhage persist, abdominal section may be resorted to as the only procedure, that offers hope.

It may not be amiss to note the "surgical leaders of the day," who defend the line of treatment thus briefly sketched:

Karl Braun, Karl Schroeder, J. Veit, August Martin, Sir Spencer Wells, T. Gaillard Thomas, and Thomas Addis Emmet, all advise the expectant plan of treatment, until it has proved futile.

The paper was discussed by Drs. Etheridge, Parkes, Engert and Mergler.

*Ovariotomy.*—The President read the histories of three cases and showed the specimens. In one of the cases the patient did well for forty-eight hours after the operation, but symptoms of pyæmia then appeared, and she died on the fifth day. At the autopsy a small sponge with a string attached to it was found in Douglas's *cul-de-sac*, firmly embedded in lymph. No hemorrhage had occurred, but general peritonitis had set in, and the cause of death was quite apparent. But for the presence of the foreign body, the patient would doubtless have recovered, and it seemed almost impossible that it should have been overlooked when the final count was made. In the second case the operation was performed within a short time after an attack of peritonitis, and the patient did well. The third case was one of papillary cystoma; the patient recovered. The president wished to emphasize the following points: (*a*) Always count the sponges used in an operation; (*b*) observe absolute cleanliness; (*c*) peritonitis is not a contra-indication of either tapping or ovariotomy; (*d*) cystic fluid left in the peritoneal cavity is dangerous and likely to cause pyæmia, but blood is probably innocuous; (*e*) a rubber blanket fastened to the patient, with a central fenestra through which to operate, is useless, small tin basins for the waste being preferable, as they can readily be changed; (*f*) each case calls for special judgment and attention to every detail.

*Laparotomy for Abdominal Tumors.*—Dr. Parkes reported three cases—two of ovariotomy (successful), and one for uterine fibroma (with a fatal termination). There was nothing remarkable about the ovariotomies, but one of the cases was interesting from the fact that the patient came near losing her life by intestinal obstruction at the end of the third week. Such an occurrence was noteworthy, as serving to show that we should be on our guard against reporting "cures" prematurely. In the case of fibroma, supra-vaginal amputation of the uterus was performed. A solid rubber cord, a quarter of an inch thick, was thrown around the pedicle at the narrowest part, and drawn as tight as the operator's strength would allow, as a temporary ligature. The mass was then cut away about half an inch above the ligature, and hemorrhage was seen to be wholly prevented, but, just as he had turned aside to place the severed mass on an adjoining table, and was about to take up the pedicle for further treatment, the rubber cord rolled over the free end of the stump, and the latter fell into the pelvis, out of sight, while the blood seemed to flow in torrents from its cut surface. Finally, the stump was grasped between the thumb and fingers and drawn out of the pelvis again, and the bleeding ceased. The pedicle was then transfixed and tied in two halves. The trouble would not have occurred if a temporary clamp had been used. The pedicle was dropped, and in closing the external wound great difficulty was met with in returning the intestines within the abdominal cavity, and keeping them there while the sutures were tied. After rallying from the anæsthesia, the patient vomited a great deal for a number of hours, and, after having ceased, the vomiting returned and lasted up to the time of her death, which took place forty hours after the operation. Only a very superficial autopsy was allowed; but this sufficed to show the cause of death to have been a gangrenous condition of about six inches of the ileum. The cause of this it was difficult to explain, for no twist had been found, and no noticeable injury was done to the gut at any time.

Both the President's cases and those related by Dr. Parkes being before the meeting, Dr. Parkes stated that he was present at the operation in which the sponge had been left in the abdomen, and that he remembered the precautions that had been taken to guard against such an accident. He admired the President's frankness in placing the case on record.

Dr. Etheridge remarked, in regard to Drysdale's corpuscle, which had been mentioned in one of the cases, that it had been found in renal cysts.

Dr. R. G. Bogue thought that it would have been strange if the patient had recovered in Dr. Parkes's case of supra-vaginal amputation of the uterus, so many and so great were the difficulties met with in the operation.

Dr. Engert asked if the bowels were at any time twisted, or if their refrigeration by long exposure had had anything to do with the gangrene.

Dr. Tilley said that the accident of leaving a foreign body in the abdomen happened oftener in this country than in Europe, because we had different assistants at different operations, whereas European operators always had the same assistants. The sponges should certainly be counted, or pieces of tape be attached to them. Regarding the confinement of the bowels for eleven days after ovariotomy, he did not think it judicious.

Dr. J. J. M. Angear remarked upon the lessons that were to be learned from cases in which mistakes were made, and on the President's conscientiousness in reporting the case of death from a sponge having been left in the abdomen.

Dr. Liston H. Montgomery and Dr. Parkes dissented from the statement that foreign bodies were oftener left in the abdomen in this country than elsewhere.

